# Posterior reversible encephalopathy syndrome in a child with cyclical vomiting and hypertension: a case report

**DOI:** 10.1186/1752-1947-5-137

**Published:** 2011-04-06

**Authors:** Zakareya Gamie, Akheel Rizwan, Frances G Balen, Michael Clarke, Mohammed M Hassoon

**Affiliations:** 1 Department of Paediatrics, Pontefract General Infirmary, Pontefract, UK; 2 Department of Radiology, Pinderfields General Hospital, Wakefield, UK; 3 Department of Paediatric Neurology, Leeds General Infirmary, Leeds, UK

## Abstract

**Introduction:**

Posterior reversible encephalopathy syndrome is characterized by headache, nausea and vomiting, seizures and visual disturbances. It has certain characteristic radiological features, which allow diagnosis in the appropriate clinical setting and enable appropriate clinical therapy to be instituted.

**Case presentation:**

A 10-year-old Caucasian girl who was hospitalized due to recurrent vomiting was diagnosed as having posterior reversible encephalopathy syndrome after an initial diagnosis of cyclical vomiting and hypertension was made.

**Conclusion:**

Posterior reversible encephalopathy syndrome is a rare disorder in children. Early recognition of characteristic radiological features is key to the diagnosis as clinical symptoms may be non-specific or mimic other neurological illnesses. To the best of our knowledge this is the first case to report an association between posterior reversible encephalopathy syndrome, cyclical vomiting and hypertension. Furthermore, in this case, the resolution of the abnormalities found on magnetic resonance imaging over time did not appear to equate with clinical recovery.

## Introduction

Posterior reversible encephalopathy syndrome (PRES) is characterized by headache, nausea, vomiting, seizures and visual disturbances [1]. PRES is commonly associated with a sudden increase in blood pressure (BP) [1]. The MRI findings have been well characterized and include vasogenic edema in the white matter of the posterior regions of the cerebral hemispheres, particularly in the parieto-occipital regions [2]. PRES is more commonly reported in adults. The cause of PRES is thought to be multi-factorial, and it may develop in patients who have hypertension, renal disease, or who are immunosuppressed [1,3]. PRES is usually reversible and prompt recognition is important [1]. In the pediatric population, PRES has been associated with chronic renal disease [4], the administration of chemotherapeutic agents [5], adrenocortical disease and Cushing's syndrome [6].

We present the case of a 10-year-old girl found to have PRES in association with cyclical vomiting and hypertension.

## Case presentation

A 10-year-old Caucasian girl presented to our department with ongoing symptoms of vomiting, non-specific abdominal pain and hypertension. She had been admitted about 15 times over a three year period with episodic attacks of frequent and severe vomiting lasting for a few days. During some of her admissions she demonstrated neurological and autonomic signs and symptoms such as confusion, disorientation, occipital headache, visual impairment, staring look, lack of response, head and eye turning to one side with nystagmus, non-reactive pupils, left arm and leg stiffening, and fluctuating and raised BP.

**Figure 1:**
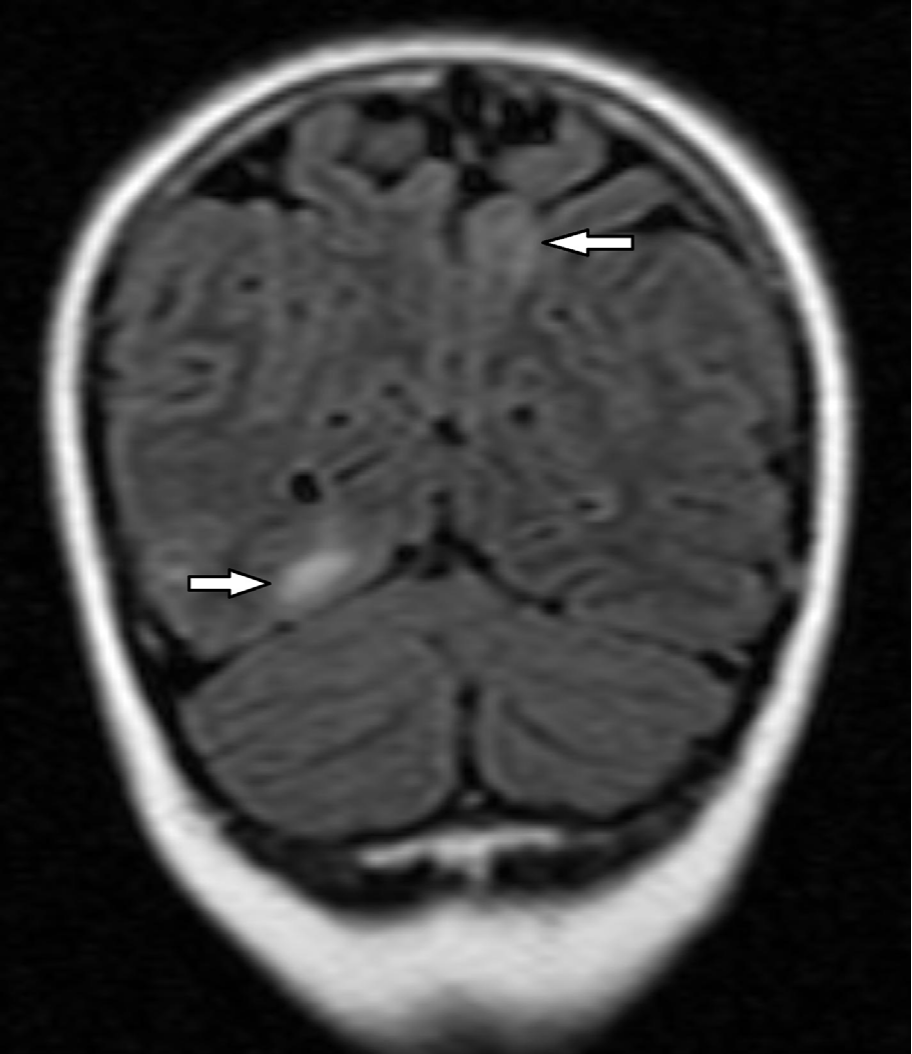
**Coronal fluid attenuation inversion recovery (FLAIR) MRI through the posterior brain showing bilateral patchy areas of high signal within the subcortical white matter of right occipital lobe and left parietal lobe**.

**Figure 2:**
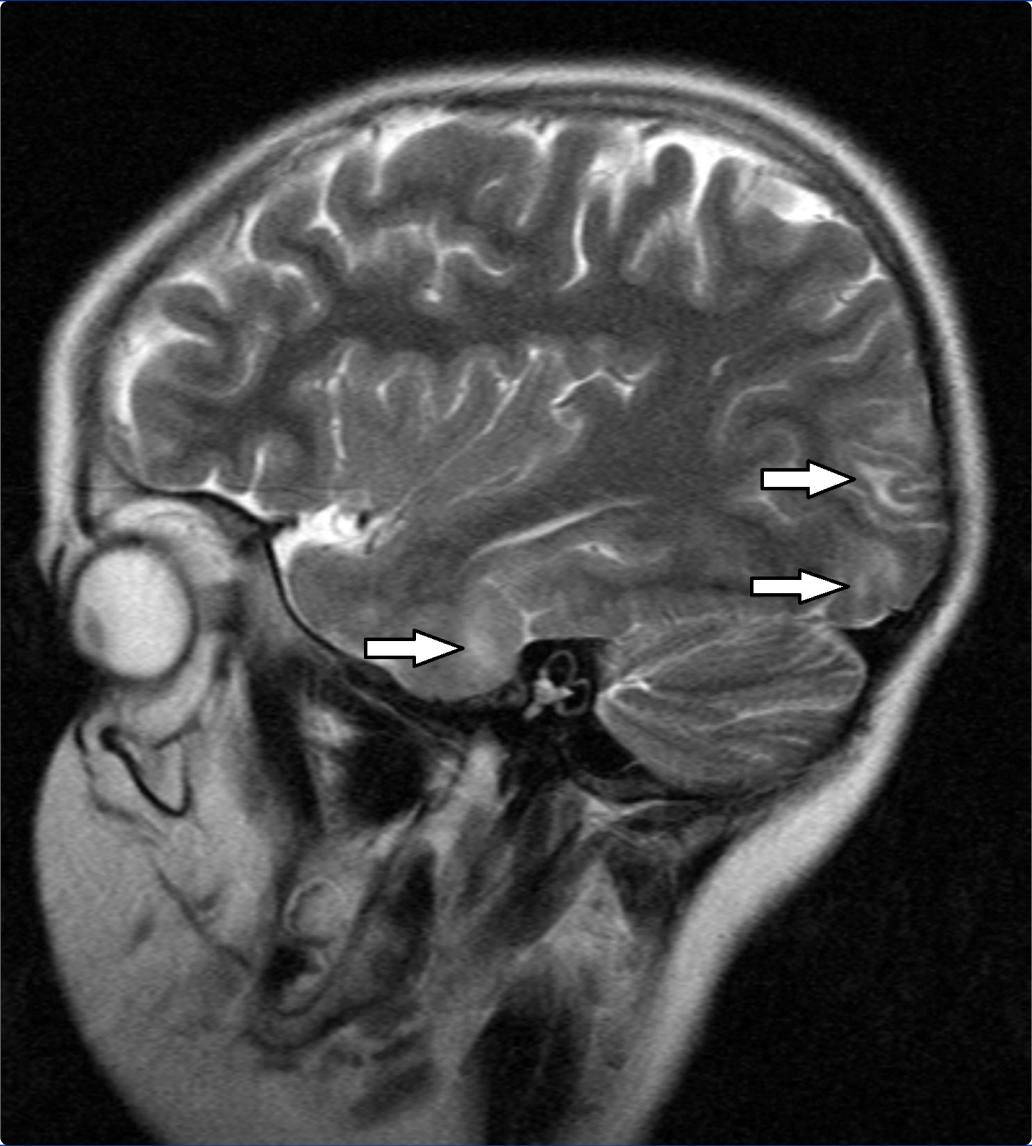
**T2 sagittal MRI through a right paracentral position showing multiple subcortical white matter lesions in the right temporal lobe anteriorly, and the right occipital lobe posteriorly**.

Investigations included abdominal and chest radiographs, and abdominal and renal ultrasonography, which all gave negative results. Gastroscopy and barium meal studies did not reveal any abnormalities, but a urease breath test revealed *Helicobacter pylori*, which was treated with standard triple therapy. A cranial computed tomography (CT) scan did not reveal any abnormalities; however, a magnetic resnonace imaging (MRI) scan (Philips Intera 1.5T) demonstrated patchy areas of mainly subcortical high signal without mass effect, contrast enhancement or associated diffusion restriction. These abnormalities were bilateral but asymmetrical, affecting the right cerebral hemisphere more than the left side. The high signal lesions were mainly located in the posterior brain, particularly the parieto-occipital lobes. No abnormality was seen in the posterior fossa or the basal ganglia. The radiological features were consistent with a diagnosis of PRES (Figures 1 and 2). An abdominal MRI was unremarkable.

Electroencephalographic (EEG) studies initially demonstrated marked right hemisphere slow wave disturbances; however, repeat studies showed no definite epileptiform abnormality, with slow and asymmetrical, frequent theta and slow activity consistent with non-specific focal organic disturbance of cerebral activity. The results of 24-hour BP monitoring (38 readings in total) revealed 10 systolic readings >118 mmHg (95th centile). Electrocardiographic (ECG) studies were also unremarkable. Midnight and morning cortisol levels were within the normal ranges. Other investigations such as urinary catecholamines, serum amylase, lactate, ammonia, cholesterol, chloride, and bicarbonate were all within normal ranges. Other investigations with normal outcomes included tests for urine porphyrin, anti-mitochondrial antibodies (AMA), anti-nuclear antibodies (ANA), double-stranded DNA (dsDNA), anti-smooth muscle antibodies (ASA), liver-kidney microsomal (LKM) antibodies, gastric parietal cell antibodies, carnitine and acylcarnitines, and a coeliac screen. A screen for orotic acid was also normal. No abnormality was detected in the urinary organic acids, but there was a slight increase in the urine amino acids. Investigations for porphyria were also normal.

Our patient was treated with the antiemetics ondansetron and cyclizine, and a trial of lorazepam was also given to try and abort the vomiting cycle. Electrolyte abnormalities were treated using intravenous fluids. At five months after her initial MRI, a repeat scan was performed and all the abnormal features had resolved. Her seizure-like symptoms settled and the vomiting episodes became shorter and less frequent. She continued to have ongoing symptoms of acute episodes of vomiting associated with hypertension for a further three months. Treatment included ondansetron, atenolol and clarithromycin. Her symptoms eventually settled and she has remained symptom free for a period of about 6 months.

## Discussion

PRES is a disorder of cerebrovascular autoregulation with multiple underlying etiologies and it is commonly associated with increases in BP [1]. In the pediatric population, PRES has been associated with chronic renal disease [4], the administration of chemotherapeutic agents [5], adrenocortical disease and Cushing's syndrome [6]. It is thought that the sudden elevation in blood pressure leads to disruption of the autoregulatory mechanisms in the central nervous system, vasodilatation and vasoconstriction resulting in a breakdown of the blood-brain barrier [5]. However, it is documented in some cases, particularly in the pediatric population, that BP may be only minimally elevated or fluctuant during the development of PRES [7].

The diagnosis of PRES can be made via CT, but MRI is a more sensitive imaging modality. The radiological appearance of PRES does not seem to be influenced by the predisposing factor [2]. The most common abnormalities on CT and/or MRI scans are focal regions of vasogenic edema involving the white matter in the posterior cerebral hemispheres, often asymmetrically and most commonly involving the parieto-occipital lobes bilaterally, often in a watershed-type distribution. The medial occipital lobe structures are spared, which distinguishes PRES from bilateral posterior cerebral artery infarcts. The posterior predilection of this condition has been ascribed to the fact that these vascular territories are sparsely innervated with sympathetic nerves [7].

Lesions that are high signal on T2-weighted fluid attenuated inversion recovery (FLAIR) sequences can also be seen in the frontal lobes, the temporal-occipital lobe and the basal ganglia and cerebellum. Patchy grey matter involvement is also recognized. MRI diffusion-weighted imaging (DWI) demonstrates that the areas of abnormality represent vasogenic edema, which is usually completely reversible once therapy is instituted [7]. Rarely, contrast enhancement can occur, presumed to reflect disruption of the blood-brain barrier. In most patients who have repeat MRI scans after correction of hypertension, there is improvement or resolution of radiological abnormalities, although hemorrhages (seen in approximately 15% of cases) can cause permanent structural damage [7].

Manifestations of PRES in the adult population include seizures, visual disturbances and headache [1]. In children, studies have also found that seizures, headache and altered mental status can be the most common clinical features [5]. The other symptoms being nausea and vomiting, and blurring of vision [5]. Studies have also revealed an altered autonomic response in patients with cyclical vomiting [8,9] and there can be a heightened sympathetic cardiovascular tone [10] and symptoms such as pallor, flushing, lethargy and fever [11]. The stress response may induce episodes of cyclical vomiting with infectious, physical or psychological stressors potentially triggering an episode [12]. It has been reported that an increased level of adrenocorticotropic hormone (ACTH) and cortisol can be associated with lethargy and hypertension before the onset of vomiting [12], and it has been hypothesized that corticotropin-releasing factor (CRF) may be a brain-gut mediator that directly connects stress and vomiting [13].

In our patient, cyclical vomiting and hypertension coexisted and were associated with PRES. Our patient was extensively investigated for endocrine, renal, gastrointestinal, neurological, cardiac and metabolic causes with no conclusive pathology. Macrolides and ondansetron were effectively used to prevent episodes of vomiting or to decrease their frequency, and this has been previously been reported in the literature [14,15]. A recent *in vitro *study has also highlighted the effectiveness of clarithromycin as a prokinetic agent [16], and in our patient it was better tolerated than erythromycin. However, more studies are required to compare the effectiveness of the two agents in patients with cyclical vomiting. Other modes of therapy include the use of tricyclic antidepressants, newer antiepileptic agents such as leveteracetam and topiramate, and the use of antimigraine medications such as sumitriptan [15]. Supportive care involves use of intravenous fluids, sedatives, analgesia and the avoidance of potential triggering factors.

## Conclusion

PRES is a rare disorder in children. Early recognition of characteristic radiological features is key to the diagnosis as clinical symptoms may be non-specific or mimic other neurological illnesses. To the best of our knowledge this is the first case to report an association between PRES, cyclical vomiting and hypertension. Furthermore, in this case, the resolution of the abnormalities found on MRI over time did not appear to equate with clinical recovery.

## Consent

Written informed consent was obtained from the patient's parents for publication of this case report and any accompanying images. A copy of the written consent is available for review by the Editor-in-Chief of this journal.

## Competing interests

The authors declare that they have no competing interests.

## Authors' contributions

ZG reviewed the literature, wrote a first draft of the manuscript, corrected, finalized and submitted the manuscript. AR reviewed the literature and edited the manuscript. FGB analyzed the MRI images, reviewed the literature and edited the manuscript. MC reviewed the literature and edited the manuscript. MH was involved with the conception of the report and managed the case. All authors read and approved the final manuscript.

## References

[B1] Lee VH Wijdicks EF Manno EM Rabinstein AA Clinical spectrum of reversible posterior leukoencephalopathy syndrome Arch Neurol 2008 65 205 210 10.1001/archneurol.2007.46 18268188

[B2] Mueller-Mang C Mang T Pirker A Klein K Prchla C Prayer D Posterior reversible encephalopathy syndrome: do predisposing risk factors make a difference in MRI appearance? Neuroradiology 2009 51 373 383 10.1007/s00234-009-0504-0 19234694

[B3] Hinchey J Chaves C Appignani B Breen J Pao L Wang A Pessin MS Lamy C Mas JL Caplan LR A reversible posterior leukoencephalopathy syndrome N Engl J Med 1996 334 494 500 10.1056/NEJM199602223340803 8559202

[B4] Onder AM Lopez R Teomete U Francoeur D Bhatia R Knowbi O Hizaji R Chandar J Abitbol C Zilleruelo G Posterior reversible encephalopathy syndrome in the pediatric renal population Pediatr Nephrol 2007 22 1921 1929 10.1007/s00467-007-0578-z 17694337

[B5] Incecik F Hergüner MO Altunbasak S Erbey F Leblebisatan G Evaluation of nine children with reversible posterior encephalopathy syndrome Neurol India 2009 57 475 478 10.4103/0028-3886.55605 19770551

[B6] Lodish M Patronas NJ Stratakis CA Reversible posterior encephalopathy syndrome associated with micronodular adrenocortical disease and Cushing syndrome Eur J Pediatr 2009 169 125 126 10.1007/s00431-009-0990-4 19415327 PMC3124700

[B7] Osborn AG Blaser SI Salzman KL Katzman GL Diagnostic Imaging: Brain 2004 Salt Lake City, UT: Amirsys

[B8] Gordan N Recurrent vomiting in childhood, especially of neurological origin Dev Med Child Neurol 1994 36 463 467 10.1111/j.1469-8749.1994.tb11873.x 8168666

[B9] Fleisher DR The cyclic vomiting syndrome described J Pediatr Gastroenterol Nutr 1995 21 Suppl 1 S1 5 8708859 10.1097/00005176-199501001-00003

[B10] To J Issenman RM Kamath MV Evaluation of neurocardiac signals in pediatric patients with cyclic vomiting syndrome through power spectral analysis of heart rate variability J Pediatr 1999 135 363 366 10.1016/S0022-3476(99)70135-6 10484804

[B11] Rashed H Abell TL Familoni BO Cardoso S Autonomic function in cyclic vomiting syndrome and classic migraine Dig Dis Sci 1999 44 74S 78S 10490043

[B12] Fleisher DR Matar M The cyclic vomiting syndrome: a report of 71 cases and literature review J Pediatr Gastroenterol Nutr 1993 17 361 369 10.1097/00005176-199311000-00005 8145089

[B13] Tache Y Cyclic vomiting syndrome: the corticotropin-releasing-factor hypothesis Dig Dis Sci 1999 44 79S 86S 10.1023/A:1026602216846 10490044

[B14] Vanderhoof JA Young R Kaufman SS Ernst L Treatment of cyclic vomiting in childhood with erythromycin J Pediatr Gastroenterol Nutr 1993 17 387 391 10.1097/00005176-199311000-00009 8145093

[B15] Li BU Misiewicz L Cyclic vomiting syndrome: a brain-gut disorder Gastroenterol Clin North Am 2003 32 997 1019 10.1016/S0889-8553(03)00045-1 14562585

[B16] Chiragh S Begum A Karim S Prokinetic effect of clarithromycin and azithromycin - *in vitro *study on rabbit duodenum Biomedica 2006 22 130 134

